# The hydrophobicity of an amino acid residue in a flexible loop of KP-43 protease alters activity toward a macromolecule substrate

**DOI:** 10.1007/s00253-020-10826-2

**Published:** 2020-08-25

**Authors:** Mitsuyoshi Okuda, Tadahiro Ozawa, Akihito Kawahara, Yasushi Takimura

**Affiliations:** 1 Biological Science Research, Kao Corporation, 1334 Minato, Wakayama, Wakayama 640-8580 Japan; 2 Biological Science Research, Kao Corporation, 2606 Akabane, Ichikai, Haga, Tochigi, 321-3497 Japan

**Keywords:** Subtilisin, Protein surface, Caseinolytic activity, Macromolecular substrate, Protein engineering, Surface engineering

## Abstract

**Abstract:**

KP-43, a 43-kDa alkaline serine protease, is resistant to chemical oxidants and surfactants, making it suitable for use in laundry detergents. An amino acid residue at position 195, in a unique flexible loop that binds a Ca^2+^ ion, dramatically affects the proteolytic activity and thermal stability of KP-43. In the present study, we obtained 20 variants with substitutions at position 195 and investigated how these residues affect hydrolytic activity toward a macromolecular substrate (casein) and a synthetic tetra-peptide (AAPL). At pH 10, the variant with the highest caseinolytic activity, Tyr195Gln, exhibited 4.4-fold higher activity than the variant with the lowest caseinolytic activity, Tyr195Trp. A significant negative correlation was observed between the hydrophobicity of the residue at position 195 and caseinolytic activity at pH 8–10. At pH 7, the correlation became weak; at pH 6, the correlation reversed to positive. Unlike casein, in the case of hydrolysis of AAPL, no correlation was observed at pH 10 or pH 6. Because the amino acid residue at position 195 is located on the protein surface and considered sufficiently far from the active cleft, the variation in caseinolytic activity between the 20 variants was attributed to changes in interaction efficiency with different states of casein at different pH values. To improve the enzymatic activity, we propose substituting amino acid residues on the protein surface to change the efficiency of interaction with the macromolecular substrates.

**Key points:**

• *A single amino acid residue on the protein surface markedly changed enzyme activity*.

• *The hydrophobicity of the amino acid residue and enzyme activity had a correlation*.

• *The key amino acid residue for substrate recognition exists on the protein surface*.

**Electronic supplementary material:**

The online version of this article (10.1007/s00253-020-10826-2) contains supplementary material, which is available to authorized users.

## Introduction

Proteases are enzymes widely used in a variety of industrial applications, such as detergents, food and feed production, peptide synthesis, leather processing, silver recovery, and waste management (Kalisz 1988). Bacterial alkaline serine proteases (e.g., subtilisins) are particularly favored because of their high productivity, high stability, and relatively low substrate specificity (Gupta et al. 2002). Due to their industrial importance, subtilisins are one of the most-studied model enzymes for protein engineering, and many excellent studies have been carried out to increase the catalytic activity, change the substrate specificity, and improve the stability of subtilisins under various conditions (Bryan 2000; Wells and Estell 1988).

Analyses of three-dimensional structures of subtilisins and subtilisin/inhibitor complexes revealed the residues involved in substrate binding in the active cleft (Bode et al. 1986; Hirono et al. 1984; Mcphalen et al. 1985). These residues were the first targets for protein engineering, particularly for improving the catalytic activity of the enzymes. For example, Glu156, Gly169, and Tyr217 of subtilisin BPN’ were substituted with Ser, Ala, and Leu, respectively (corresponding to subtilisin Carlsberg, which exhibits greater catalytic efficiency for synthetic peptides with neutral and hydrophobic P1 residues). The subtilisin BPN’ triple mutant exhibited increased catalytic efficiency for several P1 substrates, 3- to 31-fold higher than that of the wild-type enzyme. Furthermore, the P1 substrate preference of the triple mutant changed to that of subtilisin Carlsberg (Wells et al. 1987a, 1987b). However, these mutations in the catalytic cleft tend to result in biased substrate preference. In general, a mutant that prefers acidic P1 residues does not prefer basic P1 residues, and a mutant that prefers bulky P1 residues does not prefer small P1 residues (Estell et al. 1986; Wells et al. 1987a, 1987b).

In addition to residues in the active cleft, charged residues on the protein surface far from the active center reportedly affect catalytic activity (de Kreij et al. 2002; Thomas et al. 1985). Mutations of charged residues located ≥ 10 Å from the active center alter the catalytic efficiency toward synthetic peptides and the activity pH dependence. These effects are attributed to long-distance electrostatic interactions with residues in the active center and changes in the pKa of residues in the active center. However, improvements in catalytic efficiency toward synthetic peptides do not necessarily reflect enhanced activity for proteinaceous substrates, which have 20 different residues subject to cleavage (Taguchi et al. 2000).

KP-43, an alkaline serine protease secreted by *Bacillus* sp. KSM-KP43 (FERM BP-6532), is a member of a new subfamily of subtilisins characterized as “oxidatively stable alkaline proteases.” KP-43 has a molecular mass of approximately 43 kDa and is resistant to chemical oxidants and surfactants (Saeki et al. 2000; Saeki et al. 2002). Due to these properties, KP-43 has been incorporated into commercial laundry detergents.

In a previous study, we obtained a mutant exhibiting simultaneous increases in proteolytic activity, thermal stability, and surfactant stability via random mutagenesis (Okuda et al. 2013). We revealed that a single amino acid substitution (Tyr to Cys) at position 195 in a unique flexible loop that binds a Ca^2+^ ion markedly affects the enzyme’s function. Although the Tyr195Cys mutant exhibited enhanced proteolytic activity toward casein (a macromolecule substrate), it did not exhibit enhanced catalytic efficiency toward synthetic peptides.

In the present study, we obtained 20 variants with substitutions at position 195 and investigated how these residues affect the hydrolytic activity of KP-43 toward both casein and a synthetic peptide. We also discuss the importance of the relationship between the properties of the amino acid residues on the protein surface and the state of the macromolecule substrates.

## Materials and methods

### Bacterial strains and plasmids

The gene encoding KP-43 (GenBank ID: AB051423) was subcloned into the expression plasmid pHSP64, which consists of pHY300PLK (Takara) harboring the promoter region of the *Bacillus* sp. KSM-64 gene encoding alkaline endoglucanase, generating the plasmid pHSP-KP43 (Okuda et al. 2013). pHSP-KP43 was expressed in a protease-deficient derivative of *Bacillus* sp. KSM-9865 (FERM BP-10139).

### Site-directed mutagenesis

Site-directed mutagenesis at the amino acid residue at position 195 of KP-43 was performed using the overlapping PCR method (Ho et al. 1989). Mutagenesis PCR was carried out with pHSP-KP43 as the template using the primers is listed in Table S1 (ESM1). Two external primers, a sense primer (KP-43-*Bam*HI-F; 5’-end of the open reading frame [ORF] of KP-43 with a *Bam*HI site) and an antisense primer (KP-43-*Xba*I-R; 3’-end of the ORF of KP-43 with an *Xba*I site), were also used to amplify KP-43 mutant genes using the overlapping PCR method. Amplified DNA fragments were digested with *Bam*HI and *Xba*I and ligated into pHSP64.

### Protein expression and purification

The recombinant plasmid harboring the ORF encoding the KP-43 mutant gene was transformed into the protease-deficient derivative of *Bacillus* sp. KSM-9865 by electroporation (model Gene Pulser II; Bio-Rad) followed by selection on Luria-Bertani (LB) agar containing 1% (w/v) skim milk (Difco), 0.05% (w/v) Na_2_CO_3_, and 15 μg/mL tetracycline (Sigma). Tetracycline-resistant cells were selected as transformants and grown aerobically at 30 °C for 72 h in liquid medium composed of 8.0% (w/v) Polypepton S (Nippon Pharmaceutical), 0.5% (w/v) fish meal extract (Wako Pure Chemicals), 0.1% (w/v) yeast extract (Difco), 0.1% (w/v) KH_2_PO_4_, 0.02% (w/v) MgSO_4_·7H_2_O, 10.0% (w/v) maltose (autoclaved separately), and 20 μg/mL tetracycline.

### Purification of recombinant proteases

KP-43 wild-type and mutant enzymes were purified as follows. The fermentate was cleared by centrifugation, and 50 mL of the supernatant was dialyzed overnight in 10 mM Tris-HCl buffer (pH 7.5) containing 2 mM CaCl_2_. The retentate was applied to a DEAE-Toyopearl 650C column (Tosoh) equilibrated with the same buffer. The column was washed with the same buffer, and non-absorbed active fractions were collected and concentrated by ultrafiltration. Protein levels were determined with a DC-protein assay kit (Bio-Rad) using bovine serum albumin as the standard.

### SDS-PAGE analysis

The purity of each purified protease was assessed by SDS-PAGE analysis according to the method of Laemmli (1970**)**. A total of 150 ng of each purified enzyme was loaded onto an Any KD Mini-Protean TGX Stain-Free Gel (Bio-Rad). Chemifluorescence signals were captured using a Chemi Doc MP Imaging system (Bio-Rad). Precision Plus Protein Unstained Standards (Bio-Rad) were used as molecular weight markers.

### Enzyme assay

The effect of pH on caseinolytic activity was determined as follows. Casein (Hammerstein casein [Merck]) solution (0.3%) was prepared in 50 mM Britton-Robinson buffer (50 mM phosphoric acid/acetic acid/boric acid; with pH adjusted with NaOH) at pH values ranging from 6 to 10, and 200 μL of 0.3% casein solution was dispensed into the wells of a 96-well Flat-Bottom Assay Plate (Iwaki). After preincubation at 30 °C for 10 min in a Bio Shaker M/BR-024 (Taitec), a suitably diluted solution of enzyme sample (20 μL) was added and mixed gently by shaking in the Bio Shaker M/BR-024 at 30 °C for 15 min. The reaction was stopped by addition of 100 μL of 5% trichloroacetic acid. Next, 300 μL of the mixture was transferred into a Filter plate MultiScreen-HV (Merck Millipore) and centrifuged at 2500 rpm using a himac CF7D2 (Hitachi) to remove denatured protein. Subsequently, 200 μL of the filtrate was transferred into the wells of a UV Flat-Bottom Microtiter Plate (Thermo Fisher Scientific). The peptide concentration of the filtrate was determined by monitoring at 280 nm using an Infinite M200 microplate reader (Tecan). One unit (U) of caseinolytic activity was defined as the amount of enzyme needed to produce acid-soluble peptide equivalent to 1 μmol of L-Tyr per min.

### Determination of kinetic parameters

The synthetic peptide, N-glutaryl-L-Ala-L-Ala-L-Pro-L-Leu-*p*-nitroanilide (AAPL), was obtained from the Peptide Institute. Kinetic parameters were measured using six different concentrations of AAPL (0.3–10 mM) at 30 °C for 10 min in 50 mM borate buffer (pH 10) and 50 mM phosphate buffer (pH 6). The initial rate of AAPL hydrolysis was determined as described above. Michaelis-Menten kinetic parameters (*K*m and *k*cat) were determined from Lineweaver-Burk plots (1/[S] versus 1/v: [S], concentration of the substrate; v, velocity of the reaction) by least-squares linear regression using Microsoft Excel software.

### Tryptophan fluorescence measurements

Enzyme samples were set up in a 96-well Flat-Bottom Assay Plate (Iwaki) in a total volume of 200 μL per well. Fluorescence was measured at room temperature in 200 μL of 50 mM Britton-Robinson buffer (pH 6 and pH 10) containing 30 μM enzyme using an Infinite M200 PRO microplate reader (Tecan). Tryptophan fluorescence was excited at 285 nm (bandwidth 5 nm), and the emission was scanned from 285 to 450 nm (bandwidth 20 nm). All spectra were corrected for background fluorescence by subtracting a blank scan of the buffer.

### Circular dichroism measurements

Circular dichroism (CD) measurements were performed at room temperature using a CD spectrometer J720 (Jasco) in 10 mM Britton-Robinson buffer (pH 6 and pH 10) containing 0.1 mg/mL of enzyme. For CD measurements, a 0.1-cm light-path cuvette was used. The CD spectra were recorded over the range 190 to 260 nm with an interval of 1 nm and four accumulations of data. All spectra were corrected for background by subtracting a blank scan of the buffer. Spectral data are expressed as molar ellipticity, [θ] (deg cm^2^·dmol^−1^), based on mean amino acid residue weight (MRW) assuming an average weight for KP-43 of 104. The molar ellipticity was determined as [θ]*λ* = (θ × 100 MRW)/(cl), where c represents the protein concentration in mg/mL, l represents the light path length in cm, and θ represents the measured ellipticity in degrees at wavelength *λ*.

### Molecular simulation

All data sets were processed on a Windows 7 personal computer using the Discovery Studio software package (Biovia). Figures were prepared using a DS Visualizer (Biovia). The *Streptomyces* subtilisin inhibitor (SSI) and KP-43 combined structure were constructed according to the superimposed method using the X-ray crystallographic structure of KP-43 (PDB ID: 1WMD) and the structure of subtilisin BPN’ combined with SSI (PDB ID: 2SIC).

## Results

### Three-dimensional structure of KP-43

**Fig. 1:**
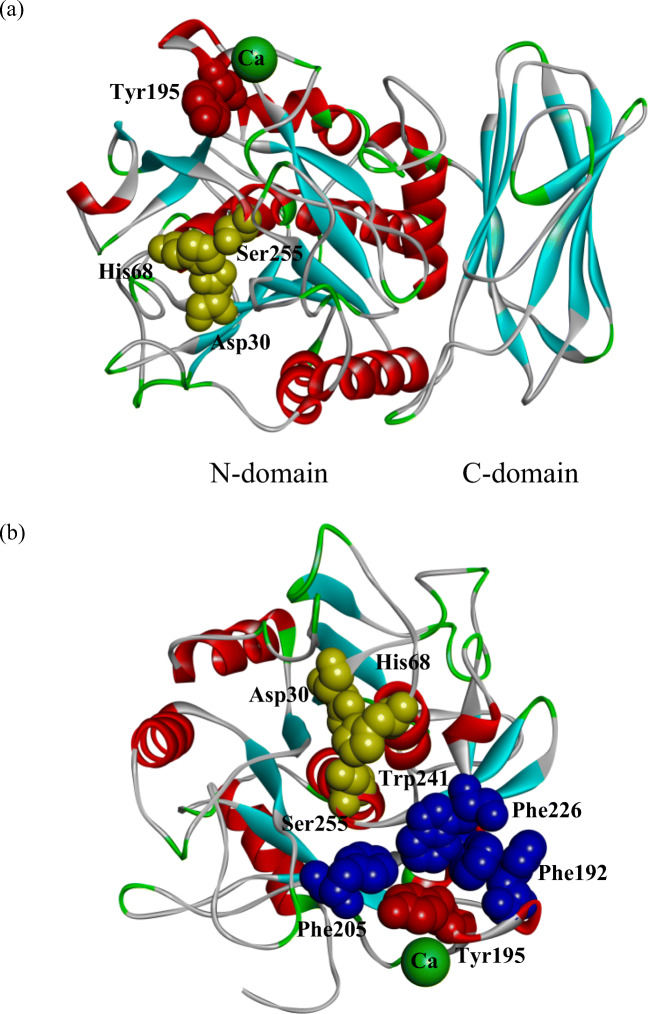
Three-dimensional structure of KP-43. **a** Three-dimensional structure of KP-43. **b** Three-dimensional structure of the N-domain of KP-43. KP-43 is shown as a ribbon representation. Calcium ion is shown as a green ball. Asp30, His68, and Ser255, comprising the catalytic triad of KP-43, are shown as CPK representations (in yellow). Tyr195 is shown as a CPK representation (in red). Phe192, Phe205, Phe226, and Trp241 are shown as CPK representations (in blue). The structures were created using DS Visualizer (Biovia)

Figure 1a shows the three-dimensional structure of KP-43 (PDB ID: 1WMD). The crystal structure indicates that KP-43 consists of two domains, a subtilisin-like α/β domain (N-domain) and a C-terminal jelly roll β-barrel domain (C-domain). Although the primary structure of KP-43 apparently differs from that of other subtilisins, the tertiary structure of the N-domain of KP-43 is highly similar to that of other subtilisins (Nonaka et al. 2004).

Tyr195 is located within a Ca^2+^-binding loop distant from the active center. The distance between Tyr195 and each residue of the catalytic triad (Asp30, His68, and Ser255) was 20 Å, 16 Å, and 15 Å, respectively. Nevertheless, the amino acid residue at position 195 affected multiple properties of the enzyme, and multiple alignment analyses indicated no amino acid residue corresponding to position 195 in other subtilisin homologs (Saeki et al. 2000).

Figure 1b shows four hydrophobic aromatic amino acid residues surrounding Tyr195: Phe192, Phe205, Phe226, and Trp241 (blue CPK representation). The distance between Tyr195 and Phe192, Phe205, Phe226, and Trp241 was 6.7 Å, 4.0 Å, 4.0 Å, and 4.4 Å, respectively.

### Caseinolytic activity of mutant enzymes at various pHs

Twenty KP-43 variants with a different amino acid substitution at position 195 were obtained and purified by column chromatography. The 20 purified variants gave a single band on SDS-PAGE analysis (Fig. S1, ESM1). All 20 variants exhibited almost the same productivity, as the coefficient of variation for the supernatant protein concentration was less than 10% for each variant (data not shown). This result indicates that the type of amino acid residue at position 195 has no effect on protein folding or protein secretion.

We measured the specific activity of each variant toward casein at pH 6, 7, 8, 9, and 10 (Table S2, ESM1). At pH 10 (the optimum pH for KP-43), the Y195W (Tyr195Trp) variant exhibited the lowest specific activity, whereas the Y195Q (Tyr195Gln) variant exhibited the highest specific activity, which was 4.4-fold higher than that of the Y195W variant. At pH 6, the Y195R (Tyr195Arg) variant exhibited the lowest specific activity, whereas the Y195I (Tyr195Ile) variant exhibited the highest specific activity, which was 1.8-fold higher than that of the Y195R variant.

**Fig. 2:**
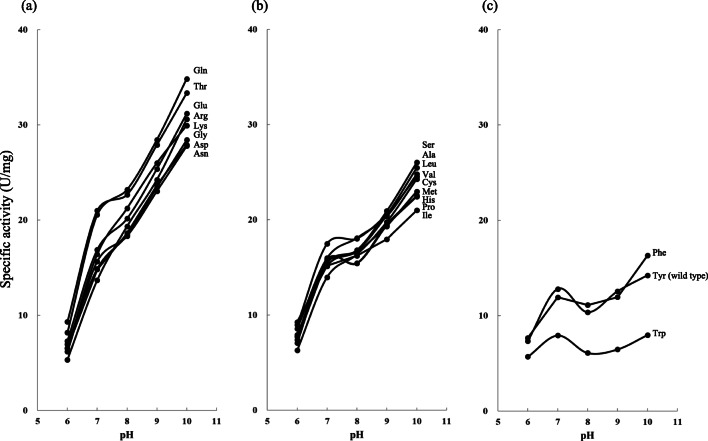
Effect of pH on the caseinolytic activity of 20 KP-43 variants. **a** Effect of pH on the caseinolytic activity of 8 high-activity KP-43 variants. **b** Effect of pH on the caseinolytic activity of 9 mid-activity KP-43 variants. **c** Effect of pH on the caseinolytic activity of 3 low-activity KP-43 variants. Enzymes were assayed at 30 °C for 15 min at the indicated pH in 50 mM Britton-Robinson buffer. Data points are plotted as the mean of two independent experiments run in duplicate

Figure 2 shows the profile of caseinolytic activity for each variant at pH ranging from 6 to 10. As shown in Figure 2a–c, three types of pH profile were observed. Figure 2a shows that 8 variants exhibited relatively high specific activity at pH 10.

Although KP-43 is an alkaline protease, wild-type KP-43 exhibited relatively high specific activity at pH 7, 90% of the activity at pH 10 (Fig. 2c). By contrast, in the case of M protease, a high-alkaline protease, the specific activity at pH 7 is only 67% of that at pH 10 (Kobayashi et al. 1995; Siezen and Leunissen 1997). Thus, high specific activity at neutral pH is a characteristic specific to KP-43.

The caseinolytic activity pH profile of each variant changed dramatically depending on the type of substitution. As shown in Fig. 2a, eight KP-43 variants (Gln, Thr, Glu, Arg, Lys, Gly, Asp, and Asn substitution at position 195) exhibited a pH profile similar to that of a high-alkaline protease. As shown in Fig. 2c, the KP-43 variant with a Phe substitution exhibited the same pH profile as that of wild-type (Tyr) KP-43. The variant substituted with Trp, another aromatic amino acid residue, exhibited specific activity lower than that of wild-type KP-43. Although KP-43 variants with hydrophobic aromatic amino acid residues at position 195 exhibited relatively low activity, their activity was generally constant, independent of pH.

The caseinolytic activity pH profiles of variants with other amino acid residue substitutions (Ser, Ala, Val, Leu, Cys, Met, Pro, His, and Ile) were between those of high-alkaline-type proteases and wild-type KP-43 (Fig. 2b).

### Effect of the hydrophobicity of the amino acid residue at position 195 on caseinolytic activity

**Fig. 3:**
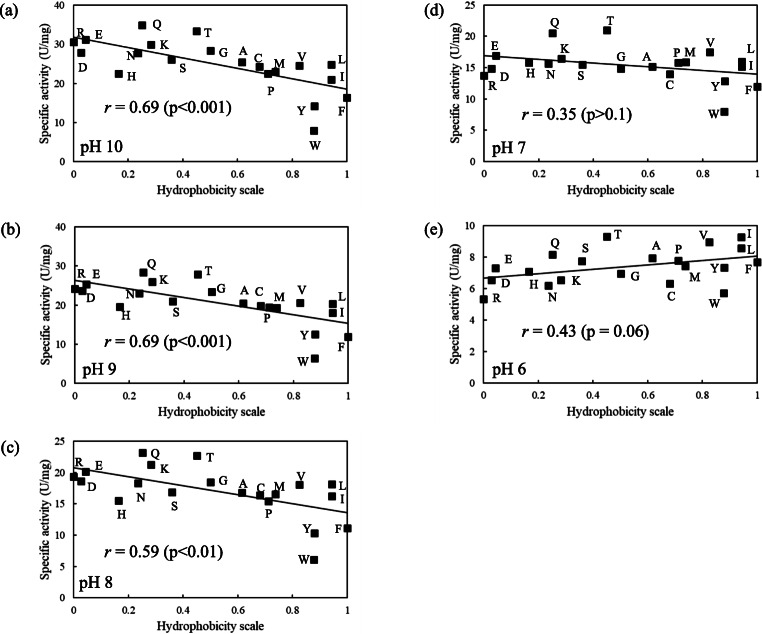
Relationship between hydrophobicity and caseinolytic activity of 20 KP-43 variants. **a**, **b**, **c**, **d**, and **e** show the relationship at pH 10, 9, 8, 7, and 6, respectively. The amino acid hydrophobicity scale was described by Black and Mould (1991). Line represents the least-squares linear regression with the correlation coefficient (*r*). Data points are plotted as the mean of two independent experiments run in duplicate

The substitution at position 195 of KP-43 had a dramatic effect on caseinolytic activity. Figure 3 shows how the hydrophobicity of the amino acid residue at position 195 affected the caseinolytic activity at various pHs. Hydrophobicity of the amino acid residues was expressed using the hydrophobicity scale values of Black and Mould (1991). Figure 3a shows the correlation between the hydrophobicity of the amino acid residue at position 195 and the caseinolytic activity at pH 10. As mentioned above, at pH 10, variants with charged amino acid residues (e.g., Glu, Asp, Arg, or Lys) or hydrophilic residues (e.g., Gln, Asn, or Thr) tended to exhibit high specific activity. Conversely, variants with hydrophobic amino acid residues (e.g., Leu, Ile, Tyr, Trp, or Phe) tended to exhibit low specific activity. As shown in Fig. 3a, the correlation was negative (correlation coefficient = 0.69; *p* < 0.001).

Because KP-43 is an alkaline serine protease, the specific activity decreases with decreasing pH. In the range of pH 10 to pH 8, the rate of decline in the specific activity of each variant was approximately 0.8–0.9 (pH 9/pH 10 and pH 8/pH 9). Thus, the positional relationship of each variant was unchanged in Fig. 3a, b, and c. The correlation coefficient was 0.69 (*p* < 0.001) at pH 9 (Fig. 3b) and 0.59 (*p* < 0.01) at pH 8 (Fig. 3c).

At pH 7, the rate of decline in specific activity (pH 7/pH 8) changed dramatically compared with either pH 9/pH 10 or pH 8/pH 9. In particular, variants with a Trp, Tyr, Phe, Pro, or His residue exhibited a rate of decline > 1.0, meaning that these variants are more active at pH 7 than pH 8. Consequently, the correlation between hydrophobicity and specific activity became weaker, as the correlation coefficient was only 0.35 (*p* = 0.12) at pH 7 (Fig. 3d).

As shown in Fig. 3e, at pH 6, although the variation in specific activity for all variants was at its minimum and the correlation between hydrophobicity and specific activity was weak, the correlation became positive, with a correlation coefficient of 0.43 (*p* = 0.06). Therefore, these results indicate that the amino acid residue at position 195 of KP-43 can have an opposite effect on proteolysis depending on pH.

### Effect of amino acid residue hydrophobicity on the hydrolysis of a synthetic peptide

To investigate the mechanism underlying the differences in proteolytic activity of the variants, we assayed the hydrolytic activity of each variant toward a synthetic peptide, AAPL, instead of the normal casein substrate. The kinetic parameters for hydrolysis of AAPL at pH 10 and pH 6 are shown in Table S3 (ESM1).

At pH 10, the Y195G (Tyr195Gly) variant exhibited the lowest *k*cat/*K*m value, whereas the Y195R (Tyr195Arg) variant exhibited the highest *k*cat/*K*m value, which was 2.5-fold higher than that of the Y195G variant. Similarly, at pH 6, the Y195G exhibited the lowest *k*cat/*K*m value and the Y195R variant the highest, which was 3.1-fold higher than that of the Y195G variant. With respect to casein proteolysis, the rank of variants by *k*cat/*K*m value was the same between pH 10 and pH 6.

**Fig. 4:**
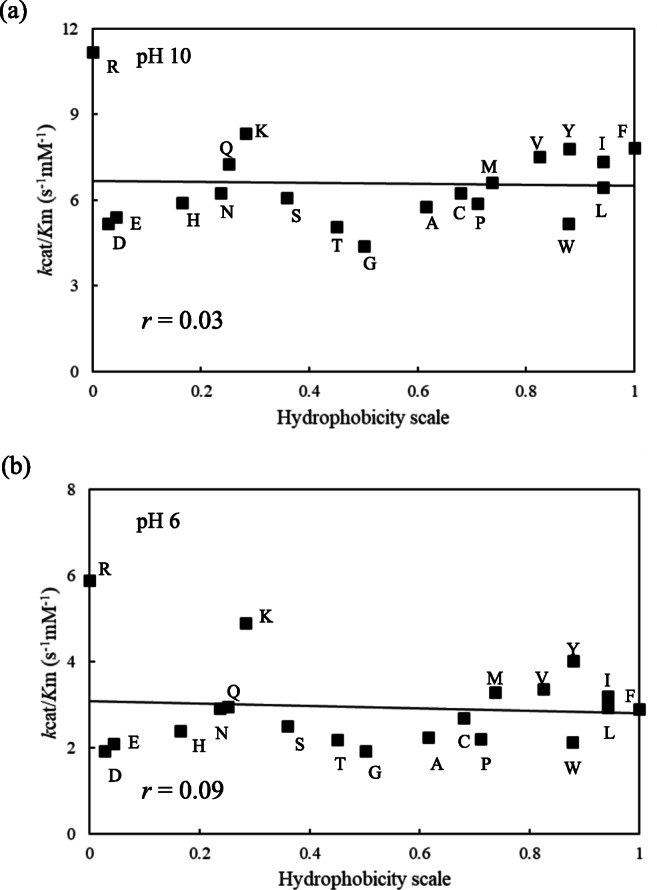
Relationship between hydrophobicity and catalytic efficiency toward AAPL of 20 KP-43 variants. **a** and **b** show the relationship at pH 10 and pH 6, respectively. The amino acid hydrophobicity scale is from the scale set described by Black and Mould (1991). Line represents the least-squares linear regression with the correlation coefficient (*r*). Data points are plotted as the mean of two independent experiments run in duplicate

Figure 4 shows how the hydrophobicity of the amino acid residue at position 195 affected the *k*cat/*K*m value at pH 10 and pH 6. Unlike the casein substrate, no correlation between hydrophobicity and *k*cat/*K*m was observed at either pH 10 or pH 6. Furthermore, the positional relationship of each variant was unchanged between pH 10 and pH 6 (Fig. 4a and b). Consequently, the amino acid substitution at position 195 had a completely different effect on AAPL and casein hydrolysis.

### Effect of the amino acid substitution at position 195 on the conformation of KP-43

To examine the effect of the amino acid substitution at position 195 on the conformation of KP-43, tryptophan fluorescence and CD measurements were carried out. As shown in Fig. 2, the 20 variants were classified into three types depending on caseinolytic activity pH profile. Y195Q and Y195S were chosen as typical variants exhibiting the highest caseinolytic specific activity at pH 10 (as shown in Fig. 2a and b, respectively).

**Fig. 5:**
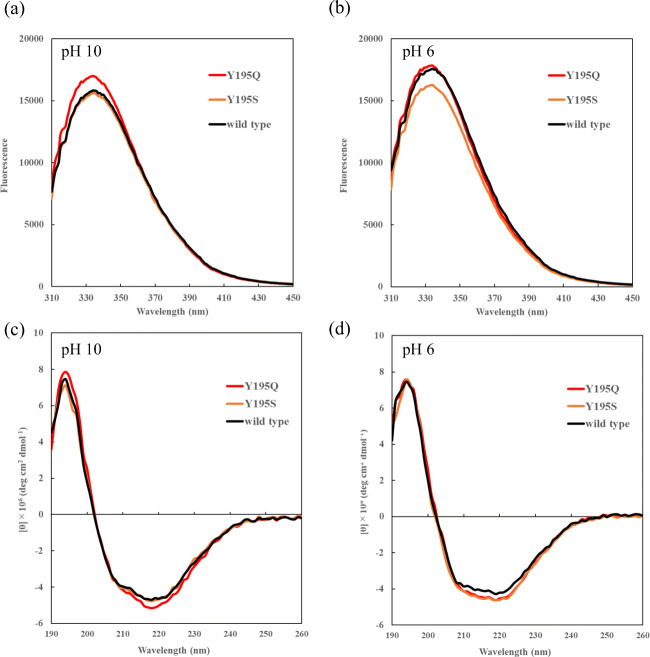
Comparison of tryptophan fluorescence spectra and CD spectra of Y195Q, Y195S, and wild-type KP-43. **a** and **b** show tryptophan fluorescence spectra at pH 10 and p H 6, respectively. **c** and **d** show CD spectra at pH 10 and pH 6, respectively. Red, orange, and black lines represent Y195Q, Y195S, and wild-type KP-43, respectively

Figure 5a and b show a comparison of tryptophan fluorescence spectra of Y195Q, Y195S, and wild-type KP-43 at pH 10 and pH 6, respectively. Y195Q, Y195S, and wild-type KP-43 exhibited maximum fluorescence intensity at 334 nm, and no peak shift was observed among the three enzymes at either pH 10 or pH 6.

Figure 5c and d show CD spectra of the three enzymes at pH 10 and pH 6, respectively. The spectra of Y195Q, Y195S, and wild-type KP-43 were almost the same, with a maximum at 194 nm and minimum at 218–219 nm, as derived from the α-helix, at both pH 10 and pH 6. These results indicate that the amino acid substitutions at position 195 had no effect on the secondary and tertiary structure of KP-43 at either pH 10 or pH 6.

### Molecular simulation of KP-43 and SSI docking

**Fig. 6:**
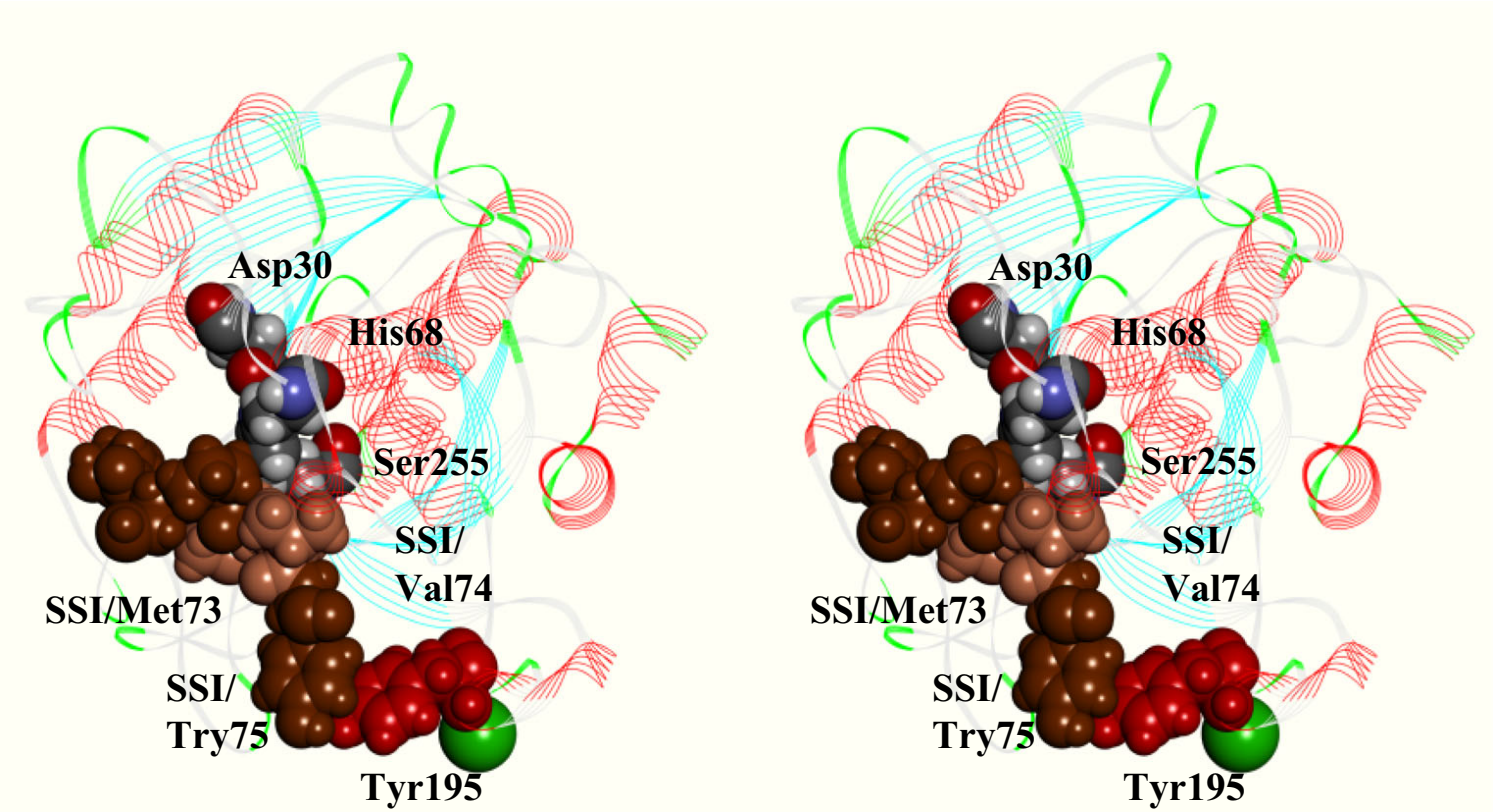
Stereo view of the structure of the N-domain of KP-43 in complex with SSI. The N-domain of KP-43 is shown as a line ribbon representation. Met70-Tyr75 of SSI is shown as a CPK representation in deep brown, Met73 of SSI (P1 residue) and Val74 of SSI (P1’ residue) are shown in light brown. Asp30, His68, and Ser255 of KP-43 are shown as CPK representations (white for hydrogen atoms, gray for carbon atoms, blue for nitrogen atoms, and red for oxygen atoms). Tyr195 is shown as a CPK representation in red. Calcium ion is shown as a green ball. The structures were created using DS Visualizer (Biovia)

Figure 6 shows a docking model of the KP-43 and SSI (PDB ID; 2SIC) complex. SSI is a very strong inhibitor of serine proteases, including KP-43 (data not shown). Previous studies indicated that Met73 of SSI is a P1 residue accommodated in the S1 site of subtilisin BPN’. Ser255 of KP-43 acts as a nucleophile that attacks the peptide bond between Met73 and Val74 (P1’ residue) of SSI (Fig. 6). The docking model suggested that Tyr195 interacts with Tyr75 of SSI (P2’ residue). The distance between Tyr195 and Tyr75 of SSI was 3.1 Å.

## Discussion

A previous study revealed that substitution of Tyr with Cys at position 195, located in the surface loop of KP-43, simultaneously increases the enzyme’s proteolytic activity and thermal/surfactant stability (Okuda et al. 2013). Many protein engineering studies have sought to improve the catalytic activity of subtilisin, revealing that amino acid residues on the protein surface, far from the active cleft, markedly affect the proteolytic activity toward macromolecular substrates (e.g., skim milk or casein).

Feller et al. (2010) investigated the relationship between protein surface electrostatic potential and specific activity toward immobilized bovine serum albumin. The authors compared the parent enzyme with chymotrypsin-like serine protease from *Cellulomonas bogoriensis* with a net charge ranging from − 2 to + 4. Positively charged mutants exhibited increased reaction rates at high ionic strength and decreased reaction rates at low ionic strength (Feller et al. 2010).

Jakob et al. (2013) substituted Asn and Gln residues on the surface of *Bacillus gibsonii* alkaline protease with Asp and Glu, respectively. The Asn253Asp and Gln256Glu double mutant exhibited increased proteolytic activity, a shift in optimum pH from 11 to 10, and twofold higher activity at pH 8.5 (Jakob et al. 2013).

Zhao and Feng (2018) used error-prone PCR random mutagenesis on the alkaline serine protease from *Bacillus pumilus* and generated 7 mutants exhibiting increased caseinolytic activity. Four of the 7 substitution sites were located on the protein surface; notably, Thr162Ile, located in a flexible loop, exhibited 2.4-fold increased caseinolytic activity (Zhao and Feng 2018). Previous studies demonstrated that both charged and uncharged amino acid residues on the protein surface can increase proteolytic activity. However, the detailed mechanism underlying the increased proteolytic activity and effect of substitutions with other amino acid residues at the mutation sites remain unclear.

Mutation sites and the effect of mutations are often discussed in reference to amino acid sequence alignments or homology models with other subtilisin homologs (e.g., subtilisin BPN’). In KP-43, Tyr195 is located in an insertion not found in other subtilisin homologs (Okuda et al. 2013). Accordingly, we could not directly compare our results with previous studies examining subtilisin mutations around position 195.

The crystal structure of KP-43 suggests that the side chain of Tyr195 is oriented out toward the solvent. Thus, the hydrophobicity of the side chain directly affects the manner of interaction with water and substrate molecules. As shown in Fig. 2, both the caseinolytic activity and pH profile changed dramatically upon substitution at position 195. It is very interesting that a single mutation in KP-43 produced three different pH activity profiles. Charged and hydrophilic amino acid residues (Gln, Thr, Glu, Arg, Lys, Gly, Asp, or Asn) rendered KP-43 a high-activity-type enzyme, whereas hydrophilic and hydrophobic amino acid residues (Ser, Ala, Val, Leu, Cys, Met, Pro, His, or Ile) rendered KP-43 a mid-activity-type enzyme. In contrast, hydrophobic aromatic amino acid residues (Phe, Tyr, or Trp) rendered KP-43 a low-activity-type enzyme.

Tyr195 is surrounded by four hydrophobic aromatic amino acid residues (Phe192, Phe205, Phe226, and Trp241) (Fig. 1b). Thus, we hypothesized that Tyr, Phe, and Trp residues at position 195 exhibit aromatic π − π interactions with these surrounding aromatic amino acid residues. The resulting restrained flexibility of the Ca^2+^-binding loop reduces the caseinolytic activity compared with other amino acid residue substitutions.

As shown in Fig. 3a–c, at pH 8–10, hydrophobicity of the amino acid residue at position 195 was negatively correlated with caseinolytic activity. However, this correlation gradually weakened with declining pH. At pH 6, the correlation between the hydrophobicity of the amino acid residue at position 195 and caseinolytic activity reversed to positive (Fig. 3e). Figure 4 shows the relationship between the hydrophobicity of the amino acid residue at position 195 and *k*cat/*K*m toward AAPL at pH 10 and pH 6. Unlike casein, no relationship was found, and the hydrolysis efficiency ranking of the 20 variants was essentially the same at pH 10 and pH 6. Furthermore, Fig. 5 suggests that there is no conformational change in KP-43 caused by the substitution at position 195. These results indicate that the observed variation in caseinolytic activity following substitution at position 195 was not due to a change in the active cleft, secondary structure, or tertiary structure of KP-43 but rather to the efficiency of the interaction with casein.

Casein, a small protein (molecular weight < 20 kDa) in milk, is an unstructured protein that forms micelles. The structure of the casein micelle has been studied for over 50 years, and various models have been proposed (Farrell Jr et al. 2006; Horne 2006). Because casein has a hydrophilic and hydrophobic domain, it exhibits amphiphilic properties. The hydrophobic core of casein micelles is surrounded by hydrophilic casein protein molecules. Liu and Guo (2008) investigated the effect of pH on the structure of casein micelles and demonstrated that the micelle structure is more compact at low pH and looser at high pH (in the range of pH 6–12) due to changes in electrostatic repulsion (casein theoretical pI is 4.8).

Figure 6 shows a docking model of the KP-43 and SSI complex, which indicates that Tyr195 interacts with Tyr75 of the SSI (P2’ residue). Consequently, the amino acid residue at position 195 likely affects the efficiency of hydrolysis due to differences in the efficiency of interaction with macromolecular substrates. Accordingly, we assume that the reversal of the correlation between the hydrophobicity of the amino acid residue at position 195 and caseinolytic activity is due to changes in the hydrophobicity of casein micelles at different pH values. However, in the case of hydrolysis of AAPL, Leu is the P1 residue, and *p*-nitroanilide is located in the position of the P1’ residue that likely does not interact with Tyr195. Therefore, substitution of Tyr195 appears to have little effect on the catalytic efficiency toward AAPL compared with the hydrolysis of proteinaceous substrates.

A number of studies have demonstrated that substitution of residues remote from the active center or substrate binding site can improve the catalytic efficiency of enzymes other than proteases. For example, Wilding et al. (2019) used a molecular dynamics approach to predict critical amino acid residues on the protein surface that interact with substrates and affect the catalytic efficiency of KES23360 transaminase. The authors demonstrated that mutation of the predicted residues (Glu178Asp, Gly179Arg, and Gln142Asn) increased the catalytic efficiency and proposed that these amino acid residues are involved in directing substrates into the active center (Wilding et al. 2018). Additionally, increasing the hydrophobicity of a specific region of the protein surface of polyester-hydrolyzing enzyme (polyesterase) increases the efficiency of polyethyleneterephthalate (PET; a hydrophobic water-insoluble substrate) hydrolysis (Biundo et al. 2018). Acero et al. (2013) conducted site-directed mutagenesis of amino acid residues on the surface of a cutinase from *Thermobifida cellulosilytica* DSM44535. Although the Arg29Asn/Ala30Val double mutant exhibited fourfold higher hydrolysis activity than the wild-type enzyme, the Gln65Glu mutant exhibited no hydrolysis of PET, regardless of the ability to hydrolyze soluble substrates (Acero et al. 2013). These results demonstrate that some residues on the protein surface are critically important for access to substrates but do not affect the structure of the active center.

The introduction of mutations on the surface of a protein is thought to be advantageous because the original substrate specificity can be retained. However, the results of this study showed that even a single mutation on the protein surface can dramatically increase proteolytic activity and differentially affect the pH profile without inducing a conformational change.

We demonstrated the importance of the relationship between the hydrophobicity of the critical amino acid residue on the surface of KP-43 and the surface properties of macromolecular substrates. We intend to investigate the relationship between the protein surface and various macromolecular substrates in more detail in order to improve enzyme activity in accordance with the features of macromolecular substrates.

## Electronic supplementary material

ESM 1 (PDF 127 kb)
