# Supplementary material for: The hydrophobicity of an amino acid residue in a flexible loop of KP-43 protease alters activity toward a macromolecule substrate
Source: Appl Microbiol Biotechnol. 2020 Aug 25;104(19):8339–49. doi: 10.1007/s00253-020-10826-2 (PMC7471176; doi:10.1007/s00253-020-10826-2)
Supplement: Supplementary file 1 — (PDF 127 kb) [file 253_2020_10826_MOESM1_ESM.pdf]

Applied Microbiology and Biotechnology

Supplementary materials

The hydrophobicity of an amino acid residue in a flexible loop of KP-43 protease alters activity toward a macromolecule substrate

Mitsuyoshi Okuda[a]*, Tadahiro Ozawa[b], Akihito Kawahara[a], Yasushi Takimura[a]

[a] *Biological Science Research, Kao Corporation, 1334 Minato, Wakayama, Wakayama 640-8580, Japan*

[b] *Biological Science Research, Kao Corporation, 2606 Akabane, Ichikai, Haga, Tochigi 321-3497, Japan*

*Corresponding author. Tel.: +81 73 426 5021; Fax: +81 73 426 5027

E-mail address: okuda.mitsuyoshi@kao.com

ORCID ID: 0000-0002-9631-7808

Table S1
Primer list

Primer name	Sequence (5' to 3')
For mutagenesis	
Y195A	CCAAGCTTTGGGTCTGCGGCGGACAATATCAACC
Y195S	CCAAGCTTTGGGTCTTCTGCGGACAATATCAACC
Y195G	CCAAGCTTTGGGTCTGGTGCGGACAATATCAACC
Y195I	CCAAGCTTTGGGTCTATCGCGGACAATATCAACC
Y195L	CCAAGCTTTGGGTCTCTTGCGGACAATATCAACC
Y195R	CCAAGCTTTGGGTCTCGCGCGGACAATATCAACC
Y195K	CCAAGCTTTGGGTCTAAAGCGGACAATATCAACC
Y195F	CCAAGCTTTGGGTCTTTCGCGGACAATATCAACC
Y195T	CCAAGCTTTGGGTCTACTGCGGACAATATCAACC
Y195E	CCAAGCTTTGGGTCTGAAGCGGACAATATCAACC
Y195D	CCAAGCTTTGGGTCTGATGCGGACAATATCAACC
Y195M	CCAAGCTTTGGGTCTATGGCGGACAATATCAACC
Y195N	CCAAGCTTTGGGTCTAATGCGGACAATATCAACC
Y195Q	CCAAGCTTTGGGTCTCAGGCGGACAATATCAACC
Y195V	CCAAGCTTTGGGTCTGTTGCGGACAATATCAACC
Y195C	CCAAGCTTTGGGTCTTGTGCGGACAATATCAACC
Y195W	CCAAGCTTTGGGTCTTGGGCGGACAATATCAACC
Y195P	CCAAGCTTTGGGTCTCCTGCGGACAATATCAACC
Y195H	CCAAGCTTTGGGTCTCATGCGGACAATATCAACC
Common reverse primer	AGACCCAAAGCTTGGGCGGAGG
For ORF of KP-43 gene cloning	
KP-43-*Bam* HI-F	AAATGGATCCGTGAGGAGGGAACCGAATGAGAAAGAAGAAAAAGGTG
KP-43-*Xba* I-R	ATATTCTAGACGATTACCATATTAATTCCTCTACCC

(a)

(b)

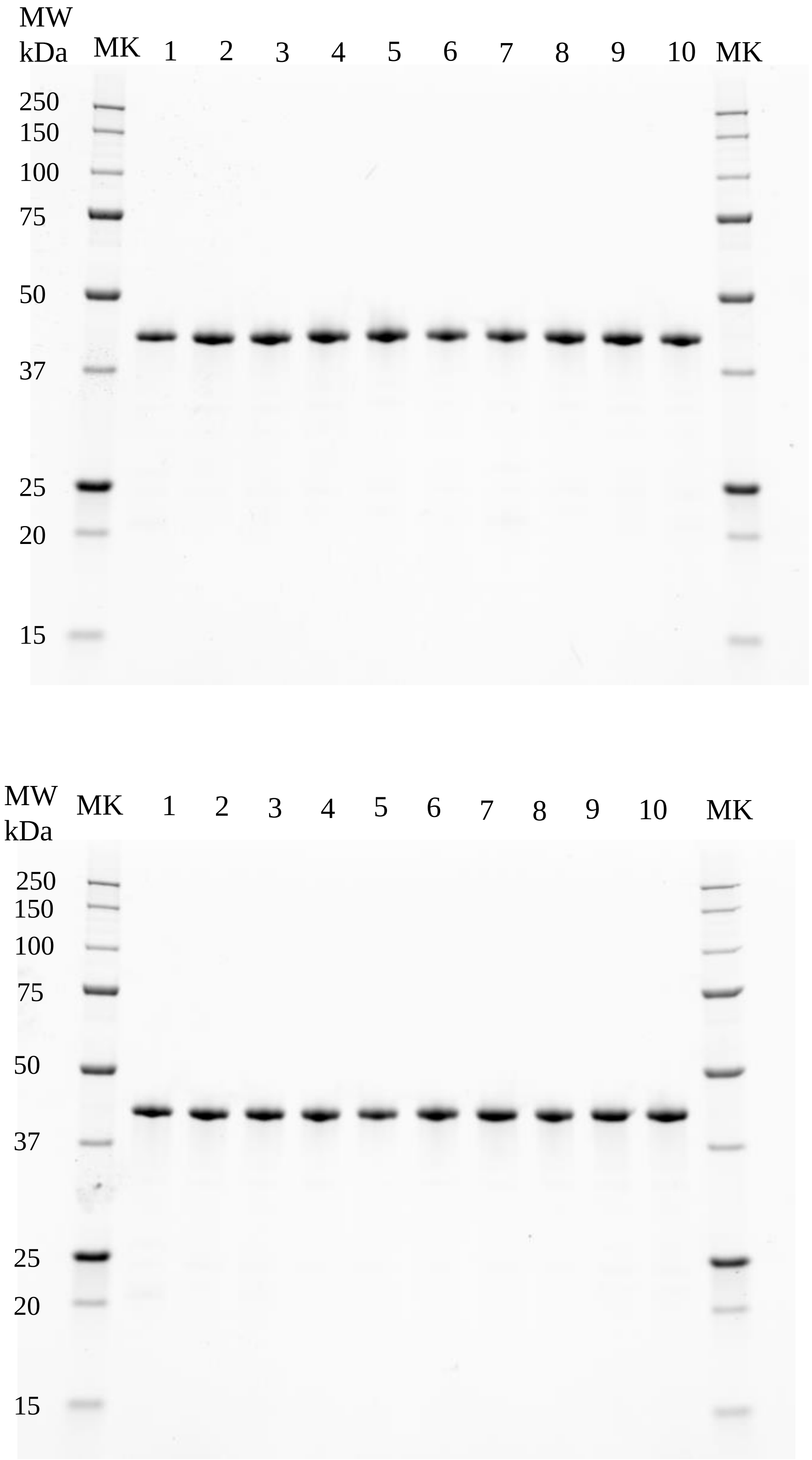

(a)
Lane1: Y195A
Lane2: Y195S
Lane3: Y195G
Lane4: Y195I
Lane5: Y195L
Lane6: Y195R
Lane7: Y195K
Lane8: Y195F
Lane9: Y195T
Lane10: Y195E

(b)
Lane1: Y195D
Lane2: Y195M
Lane3: Y195N
Lane4: Y195Q
Lane5: Y195V
Lane6: Y195C
Lane7: Y195W
Lane8: Y195P
Lane9: Y195H
Lane10: wild type

Figure S1
SDS-PAGE analysis of purified 20 KP-43 variants

Table S2
Caseinolytic activity of 20 KP-43 variants from pH 6 to 10

Residue at position 195	Hydrophobicity scale	Specific activity (U/mg)				
		pH 6	pH 7	pH 8	pH 9	pH 10
Arg	0.000	5.34 ± 0.13	13.69 ± 0.33	19.33 ± 0.29	24.22 ± 1.67	30.59 ± 1.98
Asp	0.028	6.54 ± 0.23	14.81 ± 0.51	18.62 ± 0.19	23.64 ± 1.90	27.90 ± 0.36
Glu	0.043	7.75± 0.36	16.89 ± 0.55	20.15 ± 0.41	25.37 ± 1.70	31.18 ± 1.21
His	0.165	7.08 ± 0.06	15.81 ± 0.06	15.47 ± 0.23	19.54 ± 1.54	22.53 ± 0.96
Asn	0.236	6.18 ± 0.10	15.67 ± 0.06	18.33 ± 0.06	23.04 ± 1.66	27.78 ± 0.67
Gln	0.251	8.17 ± 0.11	20.54 ± 0.53	23.18 ± 0.09	28.42 ± 1.76	34.83 ± 1.11
Lys	0.283	6.53 ± 0.16	16.41 ± 0.16	21.22 ± 0.40	25.99 ± 1.95	29.93 ± 1.50
Ser	0.359	7.75 ± 0.47	15.46 ± 0.82	16.84 ± 1.39	20.94 ± 2.47	26.04 ± 3.05
Thr	0.450	9.31 ± 0.35	21.98 ± 0.39	22.66 ± 0.19	27.90 ± 2.14	33.35 ± 1.62
Gly	0.501	6.97 ± 0.34	14.88 ± 0.80	18.46 ± 0.72	23.45 ± 2.51	28.41 ± 2.20
Ala	0.616	7.96 ± 0.44	15.16 ± 1.26	16.75 ± 1.17	20.52 ± 2.20	25.47 ± 2.92
Cys	0.680	6.31 ± 0.15	13.99 ± 0.20	16.39 ± 0.30	19.77 ± 0.69	24.28 ± 0.18
Pro	0.711	7.79 ± 0.21	15.84 ± 0.58	15.44 ± 0.85	19.39 ± 2.15	22.45 ± 1.65
Met	0.738	7.43 ± 0.19	15.86 ± 0.57	16.55 ± 0.19	19.31 ± 1.86	22.99 ± 0.95
Val	0.825	8.97 ± 0.04	17.51 ± 0.32	18.06 ± 0.19	20.60 ± 1.49	24.56 ± 0.38
Trp	0.878	5.71 ± 0.18	7.95 ± 0.18	6.10 ± 0.37	6.48 ± 0.89	7.98 ± 0.63
Tyr (wild type)	0.880	7.35 ± 0.02	12.82 ± 0.13	10.36 ± 0.19	12.57 ± 1.28	14.24 ± 0.92
Leu	0.943	8.59 ± 0.35	16.00 ± 0.72	18.11 ± 0.39	20.38 ± 1.45	24.80 ± 1.87
Ile	0.943	9.29 ± 0.15	15.20 ± 0.52	16.26 ± 0.05	17.98 ± 1.46	21.00 ± 1.40
Phe	1.000	7.68 ± 0.17	11.95 ± 0.02	11.12 ± 0.29	11.96 ± 1.09	16.33 ± 0.97

Assay was performed at 30°C for 15 min in 50 mM Britton-Robinson buffer with 0.3% (w/v) casein.

The amino acid hydrophobicity scale is from the scale set in Black and Mould (Black et al. 1991).

Data are presented as mean ± SD of two independent experiments run in duplicate.

Table S3
Kinetic parameters of 20 KP-43 variants at pH 6 and 10 toward AAPL

Residue at position 195	Hydrophobicity scale	pH 6			pH 10		
		K m (mM)	*k* cat (s^{-1})	*k* cat/*K* m (s^{-1} mM^{-1})	*K* m (mM)	*k* cat (s^{-1})	*k* cat/*K* m (s^{-1} mM^{-1})
Arg	0.000	1.51 ± 0.18	8.86 ± 0.26	5.88 ± 0.52	1.24 ± 0.05	13.83 ± 0.30	11.16 ± 0.65
Asp	0.028	2.97 ± 0.04	5.70 ± 0.37	1.92 ± 0.15	2.51 ± 0.02	12.96 ± 0.27	5.16 ± 0.06
Glu	0.043	2.51 ± 0.06	5.27 ± 0.02	2.10 ± 0.05	2.71 ± 0.09	14.64 ± 0.04	5.40 ± 0.16
His	0.165	1.70 ± 0.03	4.07 ± 0.14	2.39 ± 0.12	1.69 ± 0.14	9.98 ± 1.04	5.90 ± 0.12
Asn	0.236	1.67 ± 0.17	4.85 ± 0.03	2.91 ± 0.31	1.57 ± 0.03	9.83 ± 0.24	6.25 ± 0.04
Gln	0.251	2.05 ± 0.01	6.06 ± 0.33	2.96 ± 0.14	1.75 ± 0.34	12.61 ± 1.82	7.27 ± 0.39
Lys	0.283	1.55 ± 0.17	7.61 ± 0.52	4.90 ± 0.20	1.59 ± 0.13	13.15 ± 0.47	8.32 ± 0.39
Ser	0.359	1.82 ± 0.03	4.54 ± 0.12	2.50 ± 0.03	1.54 ± 0.08	9.33 ± 0.33	6.07 ± 0.11
Thr	0.450	2.13 ± 0.05	4.68 ± 0.28	2.19 ± 0.08	1.79 ± 0.09	9.10 ±0.77	5.07 ± 0.19
Gly	0.501	2.25 ± 0.08	4.34 ± 0.42	1.93 ± 0.12	2.16 ± 0.15	9.44 ± 0.12	4.39 ± 0.25
Ala	0.616	2.66 ± 0.23	5.96 ± 0.32	2.25 ± 0.07	1.99 ± 0.04	11.50 ± 0.67	5.76 ± 0.22
Cys	0.680	2.43 ± 0.33	6.53 ± 0.33	2.69 ± 0.23	2.57 ± 0.21	16.01 ± 0.98	6.23 ± 0.14
Pro	0.711	2.80 ± 0.18	6.16 ± 0.58	2.20 ± 0.07	1.91 ± 0.02	11.20 ± 0.33	5.87 ± 0.10
Met	0.738	1.97 ± 0.09	6.47 ± 0.20	3.29 ± 0.26	1.90 ± 0.07	12.58 ± 0.45	6.61 ± 0.02
Val	0.825	2.25 ± 0.43	7.59 ± 0.12	3.37 ± 0.06	1.77 ± 0.08	13.27 ± 0.07	7.52 ± 0.30
Trp	0.878	3.29 ± 0.19	7.01 ± 0.90	2.13 ± 0.15	1.90 ± 0.11	9.81 ± 0.11	5.17 ± 0.25
Tyr (wild type)	0.880	1.50 ± 0.20	6.02 ± 0.22	4.02 ± 0.40	1.44 ± 0.03	11.24 ± 0.02	7.78 ± 0.20
Leu	0.943	2.17 ± 0.23	6.36 ± 0.35	2.93 ± 0.15	1.85 ± 0.06	11.95 ± 0.06	6.46 ± 0.25
Ile	0.943	2.51 ± 0.54	8.02 ± 0.59	3.19 ± 0.46	1.65 ± 0.07	12.14 ± 1.28	7.35 ± 0.46
Phe	1.000	2.85 ± 0.83	8.25 ± 1.32	2.89 ± 0.39	1.53 ± 0.03	11.98 ± 0.26	7.82 ± 0.34

Assay was performed at 30°C for 10 min in 50 mM borate buffer (pH 10) and 50 mM phosphate buffer (pH 6) using AAPL as a substrate.

The amino acid hydrophobicity scale is from the scale set in Black and Mould (Black et al. 1991).

Data are presented as mean ± SD of two independent experiments run in duplicate.
